# Metabolic responses to high glycemic index and low glycemic index meals: a controlled crossover clinical trial

**DOI:** 10.1186/1475-2891-10-1

**Published:** 2011-01-05

**Authors:** Paula G Cocate, Letícia G Pereira, João CB Marins, Paulo R Cecon, Josefina Bressan, Rita CG Alfenas

**Affiliations:** 1 Departament of Nutrition and Health, Federal University of Viçosa, Avenida PH Rolfs, s/n, Viçosa, Minas Gerais, 36570-000, Brazil; 2 Departament of Physical Education, Federal University of Viçosa, Viçosa, Minas Gerais, Brazil; 3 Departament of Computer Science, Federal University of Viçosa, Viçosa, Minas Gerais, Brazil

## Abstract

**Background:**

The consumption of low glycemic index (LGI) foods before exercise results in slower and more stable glycemic increases. Besides maintaining an adequate supply of energy during exercise, this response may favor an increase in fat oxidation in the postprandial period before the exercise compared to high glycemic index (HGI) foods. The majority of the studies that evaluated the effect of foods differing in glycemic index on substrate oxidation during the postprandial period before the exercise are acute studies in which a single meal is consumed right before the exercise. The purpose of this study was to investigate the effect of consuming two daily HGI or LGI meals for five consecutive days on substrate oxidation before the exercise and in the concentrations of glucose, insulin and free fatty acids before and during a high intensity exercise.

**Methods:**

Fifteen male cyclists, aged 24.4 ± 3.8 years, with body mass index of 21.9 ± 1.4 kg.m^-2 ^and a V_O2 max _of 70.0 ± 5.3 mL.kg^-1^.min^-1^, participated in this crossover study. All test meals were consumed in the laboratory. On days 1 and 5, substrate oxidation (30 minutes before and 90 minutes after breakfast (HGI or LGI)) and diet-induced thermogenesis (90 minutes postprandial) were assessed before the exercise. The levels of glucose, insulin, and free fatty acids were determined during 2 h after breakfast on these same days. Ninety minutes after breakfast, subjects completed a 30 min cycloergometric exercise at 85 to 95% of their maximum heart rate, during which lactate concentrations were assessed.

**Results:**

The consumption of HGI meals resulted in higher areas under the glycemic and insulinemic curves in the postprandial period. However, glycemia did not differ by study treatment during exercise. There were no differences in free fatty acids in the postprandial period or in lactate levels during exercise. LGI meals resulted in lower fat oxidation and higher carbohydrate oxidation than the HGI meal in the postprandial period.

**Conclusions:**

The results do not support a differential glycemia according to glycemic index during exercise. The ingestion of LGI foods did not lead to higher fat oxidation relative to the ingestion of HGI foods.

**Trial registration:**

ACTRN: ACTRN12609000522213

## Background

The ingestion of carbohydrates in adequate amounts before exercise might be important to ensure the maintenance of glycemia during exercise [1]. In addition to their quantity, the quality of carbohydrates may be important. Glycemic index (GI) is a tool that can be used to classify carbohydrate-containing foods according to the glycemic response. It is defined as the area under the glycemic response curve after consumption of 50 or 25 g of available carbohydrate from a test food [2]. GI values are expressed relative to the glycemic response observed after the ingestion of the same amount of a reference food (glucose or white bread) [2,3].

Some authors have reported that the sharp increase in insulin secretion after the consumption of high glycemic index (HGI) foods leads to subsequent hypoglycemia and to a reduction in the availability of substrates that could be used as an energy source during exercise [4]. In contrast, the ingestion of low glycemic index (LGI) foods before exercise results in a lower and more stable glycemic response, maintaining a favourable level of glucose that can be used continuously as an energy substrate during exercise [5,6].

The results of several previous studies have indicated that the consumption of LGI foods maintains plasma glucose concentrations [4,7-9], favoring an increase in fat oxidation [4,7]. However, it is possible that substrate oxidation may depend on the type of LGI carbohydrate consumed. For instance, despite being a LGI carbohydrate, the results of a study [6] indicate that fructose consumption did not affect fat oxidation in the postprandial period before the exercise.

According to some authors there is a relationship between fat oxidation rate and lactate levels [10,11]. However, the effect of GI on lactate levels during exercise is still debatable. Although some investigators [12] have indicated that lactate levels were significantly higher with HGI feeding, others have failed to detect differences in lactate concentrations with feedings of foods varying in GI [4,6].

Most the studies published so far assessed the effect of GI of a meal consumed before a moderate intensity exercise on metabolic and biochemical responses. To our knowledge there is only one study published [13] that assessed the acute effect of GI of a meal consumed before a high intensity interval exercise. Thus, considering that the benefits of 20-30 minutes daily exercise are well established in the literature [14] and the lack of longer duration studies in which the effect of GI was assessed we evaluated the effect of the consumption, for 5 consecutive days, of two daily meals differing in IG on the metabolic responses before a high intensity intermittent exercise and the biochemical responses before and during that exercise.

The majority of the studies that have evaluated the effect of foods differing in GI on substrate oxidation during the postprandial period before the exercise and in the levels of glucose and free fatty acids before and after the exercise are acute studies in which the meal was consumed immediately before the exercise [8,9,15,16]. Substrate oxidation and the biochemical response (glucose, insulin and free fatty acids concentrations) may differ when foods varying in GI are consumed for several consecutive days. The investigation of this type of effect is not well documented in the literature. The present study was conducted to test the hypothesis that consuming two daily LGI meals 90 minutes pre-exercise for five consecutive days would lead to decreased fat oxidation in the post prandial period before exercise, when compared to the consumption of HGI meals. We also hypothesised that the LGI meals would lead to lower plasma lactate concentrations during exercise, higher free fatty acids levels, and more stable glycemic as well as insulinemic responses before and during exercise than the HGI meals.

## Methods

### Determination of the glycemic index of test meals

The selection of the types of foods served in the test meals was based on their published GI values [17]. The GI of these meals was verified through a pretest with seven normoglycemic and non-diabetic subjects (two men and five women) who were not a part of this study. These subjects had no family history of diabetes or of glucose intolerance and did not use any medications that affected glycemia. These subjects were (mean ± SD) 22.8 ± 3.1 years old and had a body mass index (BMI) of 21.4 ± 2.5 kg.m^-2^.

Subjects consumed within 15 minutes a portion of the test meal or of the reference food (glucose) containing 50 g of available carbohydrates. While test meals were consumed once, glucose was ingested three times by each participant. Capillary finger-stick blood samples were taken in the fasting state (0 min) and 15, 30, 45, 60, 90, and 120 min after the start of the test meal [18].

Glucose levels were measured using a *One Touch Ultra*^® ^glucometer. The positive area under the curve (AUC) changes in blood glucose was computed by the trapezoidal method. The AUC for each meal's glycemic response was then expressed as a percentage of the mean response from glucose consumed by the same subject [19]. The mean GI value for the test meals was determined from the GI values obtained from all seven subjects [18,19].

### Subjects

Fifteen male cyclists aged 24.4 ± 3.8 years, with mean BMI of 21.9 ± 1.4 kg.m^-2^, body fat of 7.7 ± 2.5%, and V_O2max _of 70.0 ± 5.3 mL.kg^-1^.min^-1 ^were recruited through public advertisements. Given that previous studies in males have shown that one's glycemic response to foods is affected by one's fitness status [20,21], in the present study, we selected male participants who were accustomed to routine, vigorous exercise at least three times a week. These subjects did not consume alcohol, were nonsmokers, were not using medication, were not on a therapeutic diet, had normal blood pressure, and had dietary restraint scores ≤ 14 based on a restraint scale [22].

### Study design

This controlled crossover clinical trial involved two five-day experimental sessions in which subjects were randomly assigned to consume either HGI or LGI meals for breakfast and lunch. Breakfast was served from days 1 through 5 and afternoon meal from days 1 through 4. Both test meals were consumed in the laboratory. The two five-day study sessions were separated by a washout period of at least one week.

**Figure 1:**
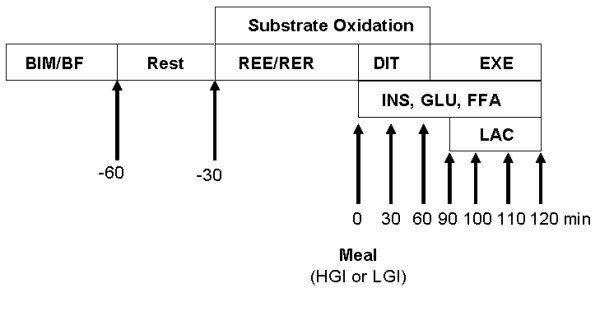
**Flow chart of experimental design**. Resting energy expenditure (REE) and respiratory exchange ratio (RER) were evaluated for 30 min before each treatment on day 1 and day 5 of each experimental session. Diet induced thermogenesis (DIT) was assessed for 90 min after the consumption of either a high glycemic index (HGI) or low glycemic index (LGI) breakfast. Substrate oxidation was determined during REE, RER and DIT assessments. Subjects exercised (EXE) for 30 minutes at 85% to 95% *V*O2max. Blood insulin (INS), glucose (GLU), free fatty acids (FFA) were determined in the fasting state (0 min) and 30, 60, 90, 120, 130, 140 and 150 min after the consumption of test meals. Blood lactate (LAC) level was evaluated immediately before the exercise (90 minutes after the consumption of test meal) and at the 10, 20 e 30-min timepoints during the exercise.

On the first and last days of each study session, subjects reported to the laboratory after a 12-h overnight fast for breakfast and evaluation of BMI, body fat percentage (Tanita^®^, TBF-300A), respiratory exchange ratio, diet-induced thermogenesis (DIT), and substrate oxidation rate. Ninety minutes after breakfast, subjects completed a 30 min cycloergometric exercise at 85 to 95% of their maximum heart rate. Venous blood samples were collected to determine glucose, insulin, and fatty acid levels in the fasting state (0 min) and 30, 60, 90, 120, 130, 140, and 150 min after the consumption of the test meals. Blood lactate levels were evaluated immediately before the exercise (90 min postprandial) and 10, 20, and 30 minutes during the exercise. A flow chart of the experimental design of the study is shown in Figure 1.

During the study, subjects received a list of HGI and LGI foods and were instructed to preferentially consume the foods having a GI similar to the GI session in which they were participating. Subjects were asked to maintain a constant level of physical activity during the study. The laboratory temperature and relative humidity varied from 24 to 26°C and 70 to 75%, respectively, during the study. The study protocol was approved by the Human Subjects Review Committee of Federal University of Viçosa-MG, Brazil (Of.Ref.N° 021/2006/Comitê de Ética).

#### Maximal cardiac frequency assessment

A week prior to the beginning of the study sessions, the maximal oxygen uptake (V_O2 max_) of each subject was estimated during an incremental cycling exercise to volitional fatigue, using an electronically-braked cyclo ergometer (Ergo Fit, model Ergo Cycle 167). Subjects started the bicycle test at a workload of 50 watts. Every two minutes, the workload was increased by 50 watts until the maximal workload was reached [23]. Maximal oxygen uptake and fitness [24] were estimated by taking into account the highest workload (in watts) achieved by each subject and his body weight as determined before the beginning of this test, according to the Balke, & Ware (1959) [25] protocol:

$${\text{V}}_{\text{O}2\max}\;\text{mL}\;{\left(\text{kg}.\min\right)}^{-1}=\frac{200+\left(12\times \text{work}\;\text{load}\left(\text{watts}\right)\right)}{\text{body}\;\text{weight}\left(\text{kg}\right)}$$

Based on the results obtained during maximal oxygen uptake testing, each participant's maximal heart rate (HR_max_) was then determined and used to calculate his workload intensity, using the heart rate reserve equation proposed by Karvonen Kentala & Mustala (1957) [26] for an intensity range between 85 and 95%:

$$\text{Target}\;\text{HR}={\text{HR}}_{\text{resting}}+\%\;\text{of}\;\text{the}\;\text{workload}\;\text{intensity}\left({\text{HR}}_{\text{max}}-{\text{HR}}_{\text{resting}}\right)$$

During the study sessions, patients' heart rates were assessed each minute in a standardised way.

#### Test meals

Participants consumed corn flake cereal, whole milk, sports drink (high carbohydrate, water, and electrolytes replenisher), white bread, and margarine for the HGI meal. All Bran cereal, fat-free strawberry yogurt, grape juice, multi-grain bread, margarine, and apple were provided in the LGI meal. The fiber content of test meals was adjusted by the addition of fiber supplement (Benefiber) to the HGI meal. Glucose was added to the milk consumed in the HGI group. Fructose was added to the grape juice in the LGI group.

There were no significant differences in macronutrient composition, fiber content, or energy density between the LGI and HGI meals (Table 1). Subjects were instructed to consume all the foods provided within 15 minutes. Test meals contained a total energy that was equivalent to 1/3 of each participant's resting metabolic rate, and they contained approximately 2 g of available carbohydrate per kilogram of the participant's body weight [4,6].

**Table 1:** Glycemic index (GI), weight, energy density, macronutrient composition, and fiber content of the high glycemic index (HGI) and low glycemic index (LGI) test meals

Test meal	GI	Type of sugar added to the meal (g)	Weight (g)*	Energy density (kcal/g)	(% total kcal)			Fiber (g/100 g)
						
					Carbohydrate	Protein	Fat	
HGI	79	Glucose 46.66 ± 2.24	468.00 ± 53.24	1.4	83.5	7.2	9.4	10.5

LGI	28	Frutose 21.3 ± 1.83	646.24 + 72.23	1.1	83.5	7.2	9.4	10.5

*The weight of the test meals (mean ± SD) varied according to the body weight of the subjects.

#### Energy expenditure and substrate oxidation assessment

Participants were instructed to refrain from heavy physical activity on the day preceding the tests. Subjects rested in a quiet and dark room in a supine position for 30 min before the test began [27].

Resting energy expenditure (REE) and substrate utilization were assessed in the supine position for the next 30 min through indirect calorimetry (Deltatrac II^® ^Datex, Finland). The respiratory exchange ratio was calculated as CO_2 _produced/O_2 _consumed [28,29]. Respiratory exchange ratio was converted into kilocalories of heat produced per body surface area per hour and extrapolated to total energy expenditure [28,29].

Next, subjects consumed either the LGI or HGI meal and returned to the indirect calorimeter. DIT was measured for 90 minutes. Then, subjects cycled in a cyclo ergometer at 85 - 95% of their maximum heart rate intensity, which was evaluated as described below.

Fat and carbohydrate oxidations were estimated with the V_O2 _and V_CO2 _values obtained during the REE and DIT assessments, using the following equations [30]:

$$\text{Fat oxidation rate }\left(\text{g}/\text{min}\right)=\text{1},\text{695}\;\times {\text{V}}_{\text{O}2}-1,701\times {\text{V}}_{\text{CO}2}$$

$$\text{Carbohydrate oxidation rate }\left(\text{g}/\text{min}\right)=\text{4},\text{585}\times {\text{V}}_{\text{CO}2}-3,226\times {\text{V}}_{\text{O}2}$$

#### Exercise

After a 5 min warm-up at 40% of the maximal heart rate, subjects cycled in the cyclo ergometer at 85-95% of their maximal heart rate in three sets of 10 min and intervals of 2 min between these sets. Immediately before cycling (resting period), at 5 and 10 min of each set, the heart rate was evaluated using a heart rate monitor (Polar^®^, model M31). Blood pressure was assessed using a sphygmomanometer and a stethoscope (Wan Med^®^). Subjective perceived exertion was evaluated at times 0, 5, and 10 min of each set [31]. While cycling, subjects drank 3 mL of water per kilogram of body weight after the warm-up, 5 minutes after the beginning of the second set, and in the last minute of the last set [32,33].

#### Blood sample analysis

Blood samples were drawn from an antecubital vein using disposable 10 mL BD^® ^syringes and BD^® ^PrecisionGlide needles (0.80 × 25 mm) during substrate oxidation assessments and exercise. Blood samples were centrifuged at 3,000 rpm for 10 min immediately after collection. Plasma was stored at -20°C, and analyzed 10 days later. Serum was analyzed for glucose colorimetrically using an automated sample analyzer (Cobas Mira, Roche^®^). Serum insulin was analyzed by radioimmunoassay (Linco Research^®^). Blood lactate concentrations were analyzed by the enzymatic method using the automated sample analyzer (Cobas Mira, Roche^®^). Plasma non-esterified fatty acids were analyzed using the Wako^® ^NEFA C kit.

#### Statistical analysis

A three-way repeated measures analysis of variance (ANOVA) on three factors (diet*days*minutes) was used to analyze differences in the physiological and metabolic responses (cardiac frequency; blood pressure; blood glucose, insulin, lactate, free fatty acid concentrations; respiratory exchange ratio; energy expenditure; and substrate oxidation) in both study sessions. A two-way ANOVA on two factors (diet*day) was also used to evaluate the differences in the area under the glycemic and insulinemic curves in the experimental sessions. Analyses were conducted using the SPSS software package (release 10.0.5; SPSS, Chicago, IL). The criterion for statistical significance was *p *< 0.05, and all tests were two tailed. The results are reported as means ± standard error (SE).

## Results

### Heart rate, blood pressure, and perceived exertion

There were no statistical differences (p > 0.05) in heart rate, blood pressure, and perceived exertion between subjects in the two study sessions.

### Glycemic and insulinemic responses

**Figure 2:**
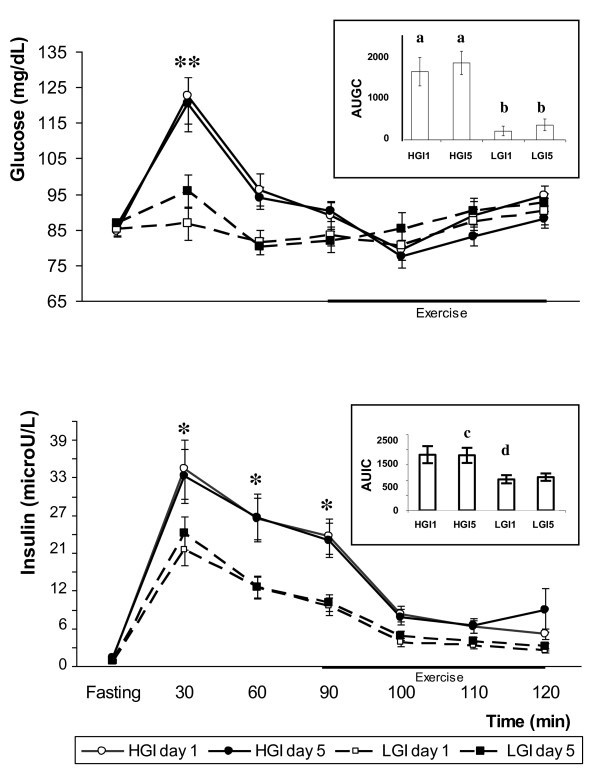
Mean ± SE fasting, postprandial, and during subsequent exercise glycemic and insulinemic responses following the ingestion of high glycemic index (HGI) and low glycemic index (LGI) meals on days 1 and 5. HGI postprandial glycemic response (**) at 30 min and insulinemic responses (*) at 30, 60, and 90 min are significantly higher than LGI response on days 1 and 5. The observed glycaemic response is significantly associated with the glycaemic index, study day and time of exercise. The inner figure represents the mean ± SE area under the glycemic (AUGC) and insulinemic (AUIC) response curves in the first and last day of each experimental session. HGI AUGC on days 1 and 5 is significantly higher than LGI AUGC (a, b). Day 5 HGI AUIC is significantly higher than day 1 LGI AUIC (c, d).

Peak glycemic and insulinemic (IR) responses were reached 30 minutes after the consumption of the test meals. These responses were significantly higher (p < 0.05) for the HGI than for the LGI meal on days 1 and 5. On both test days, the IR with the HGI meal during the 30-90 min period was significantly higher than the IR with the LGI meal in that same period of time (Figure 2).

The glycemic level 30 minutes after the ingestion of the HGI meal was significantly higher than at all the other times on both test days. On these days, glycemic response following the HGI meal at the 60-min timepoint was significantly higher than that at the 100-min timepoint. The glycemic response to the LGI meal at the 30-min timepoint was significantly higher than that at the 60-min timepoint on day 5. A significant interaction (p < 0.001) was observed among the glycemic index, the study day and the time of exercise (Figure 2).

The IRs with the HGI meal at 60 and 90 min were higher than the response during the 100 - 120 minute period on days 1 and 5. On day 1, the IR following the LGI at 60 min was significantly higher than the IR at fasting or at 110 and 120 min. On that same day, the IR with the HGI at fasting was significantly lower than the IR at 90 min.

The area under the glycemic response curve (AUGC) after the consumption of the HGI meal was significantly higher than that after the LGI meal on days 1 and 5 was. On the other hand, the area under the insulinemic response curve (AUIC) after the fifth day of consumption of the HGI meal was significantly higher than the one observed on the first day of ingestion of the LGI meal (Figure 2). There were no differences in the AUGC or the AUIC between the first and the last days within each study session.

### Lactate and free fatty acids concentrations

There were no differences (p > 0.05) in lactate or free fatty acid concentrations between the study treatments. The consumption of HGI or LGI meals for five consecutive days did not change free fatty acid concentrations from their baseline levels (day 1). The concentration of free fatty acids at baseline was significantly higher than that at 110 min after ingestion of the HGI meal on day 5.

### Substrate oxidation rate

**Figure 3:**
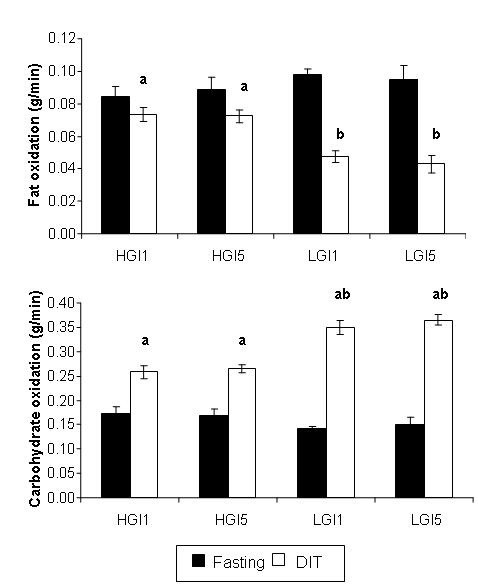
**Mean ± SE fat and carbohydrate oxidation in the fasting state and diet-induce thermogenesis (DIT) (90 min postprandial) after the ingestion of high glycemic index (HGI) and low glycemic index (LGI) breakfast meals on days 1 and 5**. Fat oxidation during HGI DIT evaluation is higher than LGI DIT (a,b) on days 1 and 5. Carbohydrate oxidation during LGI DIT evaluation is higher than HGI DIT (c,d) on days 1 and 5.

Fasting fat and carbohydrate oxidation rates were similar (p > 0.05) in both experimental sessions. However, during the DIT evaluation (90 min postprandial), fat oxidation rate was higher (p < 0.05) on days 1 and 5 of the HGI than on those days of the LGI session. The fat oxidation rate for the fasting state was higher than during the DIT evaluation after the LGI meal on these same days. The fasting carbohydrate oxidation rates were lower than those at the 90 min postprandial period on days 1 and 5 of both study sessions. There was a higher carbohydrate oxidation rate in the postprandial period (DIT) on days 1 and 5 after the ingestion of the LGI meal than there was after the ingestion of the HGI meal (Figure 3).

### Respiratory quotient

**Figure 4:**
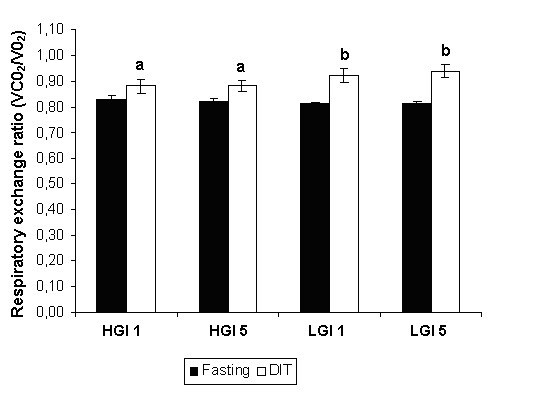
**Mean ± SE respiratory exchange ratio in the fasting state and during 90-min diet induce thermogenesis (DIT) after the ingestion of high glycemic index (HGI) and low glycemic index (LGI) breakfast meals on days 1 and 5 of each experimental session**. HGI DIT values are lower than LGI DIT values (a,b).

The study treatment did not affect the respiratory exchange ratio evaluated in the fasting state or during DIT evaluation. The respiratory exchange ratio with fasting was lower than that during the DIT assessment on both days of the HGI and LGI sessions. There were significantly higher DITs on days 1 and 5 of the LGI session than on the same days of the HGI session (Figure 4).

## Discussion

Lower glycemic and insulinemic responses were observed when two daily LGI meals were consumed for 5 consecutive days compared to the consumption two daily HGI meals for this same period of time. However, these responses did not differ according to GI during exercise. There was a lower level of fat oxidation in the 90 minutes pre-exercise after the ingestion of the LGI meal than there was after the ingestion of the HGI meal. GI did not affect free fatty acids levels before and during exercise. Lactate concentration during exercise was also not affected by the study treatments.

It has been suggested that the ingestion of LGI foods, compared to HGI foods, results in lower and more stable glucose and insulin levels [3,4,6]. However, the consumption of breakfast cereals differing in GI did not lead to differences in the areas under the insulinemic response curves between the LGI and HGI groups in our study. In one prior study, the lower GI seen after consumption of bran cereal was attributed to an earlier postprandial hyperinsulinemia, which attenuated the increase in the plasma glucose concentration [34]. However, the bran cereal in that study had almost 4 times more protein than the corn flakes (high GI cereal) [34]. It has been demonstrated that the addition of protein to a meal increases insulin secretion, leading to a reduction in the GI of that meal [19,35].

In the present study, the test meals had the same protein content. The highest glycemic response was reached 30 min after the consumption of the HGI and the LGI meals. HGI glycemic levels from 60-120 min were significantly lower than the peak level. However, these levels were not lower than the baseline level (fasting condition). LGI glycemic response was stable during the 60-120 min period.

Ingestion of the LGI meal did not affect the glycemic response during the postprandial period and during exercise [5]. Although the glycemic response observed in the first day did not vary significantly, the consumption of the LGI meal for five consecutive days led to a significantly higher glycemia at 30 min than at 60 min. This result suggests that although the short-term glycemic response to the LGI meal may lead to stable glycemia, this type of response may not be maintained when LGI foods are ingested for several consecutive days. The mechanism responsible for the change in the observed glycemic response pattern should be investigated in future studies.

According to some authors, the lower and more stable glucose levels observed after the consumption of LGI foods (versus HGI foods) is important to maintaining an adequate concentration of glucose for use as an energy source during exercise [4,6]. In the present study, the 30-90 min IR was significantly higher after consumption of the HGI than after consumption of the LGI meal. However, there was no difference in this type of response during exercise. Similar responses were verified in other studies in which foods differing in GI were consumed before cycling in a cyclo ergometer at 70% of V_O2 max _[8,9,16].

There was no effect of test meals GI on free fatty acid concentrations during the 120 min in which this parameter was evaluated. However, in two other studies [5,15] there were lower free fatty acid concentrations during the 180 min after the consumption of a LGI meal compared to a HGI meal. However, fructose was not an ingredient in the test foods in these last two studies mentioned [5,15], whereas fructose was included in the LGI meals in our study.

Fructose decreases insulin secretion [36] and is rapidly taken up independently of insulin, primarily in liver cells, where it is quickly metabolized to triose-phosphates, which can be oxidized or released as lactate [37]. Due to its ability to reduce insulin secretion, fructose down-regulates lipoprotein lipase (LPL) activity [38]. LPL is a central enzyme of lipid metabolism, which catalyzes the hydrolysis of triglycerides, producing free fatty acids and glycerol [39]. Therefore, the presence of fructose in the LGI meal in this study favored the lower IR observed, which, in turn, may have reduced LPL activity, leading to a reduction in triglycerides hydrolysis and therefore in the release of free fatty acids.

However, the concentrations of free fatty acids after the consumption of HGI meals in the present study were similar to those observed in other studies [6,9,15]. In all these studies, the postprandial (before and during exercise) fatty acid levels did not change significantly with time. However, these levels were lower than the levels measured during the fasting condition. These responses were possibly due to the higher glycemic response and IR observed in this study, as well as the higher carbohydrate oxidation rate seen after the ingestion of the HGI meal than when subjects were fasting.

In the present study, as well as in others [4,6], lactate levels during exercise were not affected by GI. Diets differing in GI, when consumed before exercise by recreational athletes, did not affect the lactate concentration during a 60 min exercise at 65% of V_O2 max _[6] or 70% of V_O2 max _[4]. Therefore, the results of these studies indicate that lactate concentrations are not affected by GI in active subjects.

The results obtained in the present study and in two other studies [6,40] indicate that the ingestion of high carbohydrate meals increases the levels of carbohydrate oxidation compared to the fasting state, independently of the GI of the meal consumed. In the present study, there was a lower fat oxidation and a higher carbohydrate oxidation in the 90 min period following the ingestion of the LGI meal than there was with the HGI meal.

However, an earlier study [5] reported a 118% increase in fat oxidation during the first 80 min of exercise associated with the consumption of LGI foods compared with the consumption of HGI foods. In a more recent study [15] conducted by these same authors, it was shown that during exercise at 70% of V_O2 max _conducted for 30 min by recreational runners, there was more fat oxidation after the ingestion of LGI foods than after the ingestion of HGI foods. Although fat oxidation was not affected by the GI of test foods consumed prior to exercise in that study [15], the consumption of an LGI diet 3 h before exercise at 65% of V_O2 max _did not affect fat oxidation before the exercise [6]. In contrast, the consumption of HGI diets, resulted in higher carbohydrate oxidation during a 2-hour period of exercise at 70% of V_O2max _[9,16]. The results of these studies suggest that while substrate oxidation is not affected by GI of test meals consumed prior to exercise, the consumption of a LGI diet resulted in an increase in fat oxidation during exercise [5,6,16].

However, the lower fat oxidation observed in the present study could reflect the way in which fructose affects the oxidation of substrates. Other investigators showed that the ingestion of 50 g of fructose (LGI) caused a higher carbohydrate oxidation and a lower fat oxidation than when the same amount of glucose (HGI) was consumed [41]. This indicates that the consumption of LGI meals containing less fructose (21.3 ± 1.83 g in the present study) may also result in this same substrate oxidation profile.

Fructose metabolism occurs mainly in the liver. This carbohydrate rapidly enters the cells through GLUT 2, without any energy cost or insulin requirement. Once inside the cells, fructose is rapidly converted into fructose-1-phosphate, which can then lead to the formation of fructose-1, 6-bisphosphate. This substrate can, in turn, be converted into glucose, acting as a primary glycolysis and oxidative phosphorylation supplier (carbohydrate oxidation) [42].

Nevertheless, in another study the consumption of a low GI test meal containing about the same amount of fructose (0.37 g/kg of body weight) as used in this study (0.32 g/kg of body weight) prior to running on a motorised treadmill resulted in similar level of fat oxidation compared with the high GI meal [6]. Factors contributing to the differences between that study [6] and the present study are not well understood and should be investigated in future studies.

It has been shown that fructose consumption results in an influx of carbohydrates into cells, increasing glycolysis and mitochondrial citrate synthesis. The subsequent increase in malonyl coenzyme A inhibits the activity of carnitine palmitoyltransferase 1, which transports fatty acids into mitochondria, leading to a reduction in fat oxidation [41]. Therefore, the results of the present study suggests that the consumption of fructose 90 minutes before a competition may not be recommended because fructose favors a higher carbohydrate oxidation, which can, in turn, lead to a reduction in glycogen storage, potentially compromising the main energy substrate used during exercise.

Compared with the HGI session, during the LGI session (fructose ingestion), there was a higher respiratory exchange ratio during the DIT assessment. This result is the opposite of the results reported in two other studies [5,6]. However, in the present study and in the study by Wu et al. (2003) [6], the respiratory exchange ratio during fasting was lower than the respiratory exchange ratios after the consumption of the HGI and LGI meals. These results suggest that, compared with the fasting condition, there was an increase in postprandial carbohydrate oxidation, which was not dependent on the GI of the ingested meal.

The energy expenditure after the consumption of HGI and LGI meals on days 1 and 5 of each session was higher than that in the fasting condition. A similar response was observed after the consumption of 75 g of different sources of carbohydrate [43]. Such effects reflect the increase in energy expenditure for digestion [27], absorption [44], metabolism, and storage of the consumed nutrients, as a result of the DIT [27]. According to Westerterp (2004) [44], carbohydrates are responsible for 5-10% of the energy expenditure from DIT. However, the DIT was not affected by the GI of the test meals in the present study.

In contrast, in another study [45] the consumption of 25 g of xylitol (GI = 7) did not modify significantly the energy expenditure in the 60-120 min postprandial when compared with the fasting condition. However, the ingestion of 25 g of glucose (GI = 100) led to an energy expenditure that was significantly higher than that at baseline. According to the authors of that study, although these sugars were consumed in isocaloric portions, xylitol enters the cells independently of insulin action and is slowly converted to glucose-6-phosphate. Xylitol does not affect fat and/or carbohydrate oxidation. All D-glucose derived from xylitol metabolism is primary stored as glycogen in the liver, which is then gradually released into the blood stream as glucose [46].

## Conclusions

The consumption of a HGI meal resulted in greater areas under the glycemic and the insulinemic curves than the ingestion of an LGI meal did on days 1 and 5. However, there were no differences in the glycemic and insulinemic responses during exercise. Concentrations of lactate and free fatty acids were not affected by the GI of the meals. The HGI meal led to higher fat oxidation, while the LGI meal resulted in higher carbohydrate oxidation. Postprandial energy expenditure was not affected by the GI of the ingested meals.

## Abbreviations

AUC: area under the curve; AUIC: area under the insulinemic response curve; AUGC: area under the glycemic response curve; BMI: body mass index; DIT: diet-induced thermogenesis; FFA: free fatty acids; GI: glycemic index; HR_max_: maximal heart rate; IR: insulin response; HGI: high glycemic index; LGI: low glycemic index; PP: postprandial period; REE: Resting energy expenditure; SD: standard deviation; SE: standard error.

## Competing interests

The authors declare that they have no competing interests.

## Authors' contributions

RCGA: study conception and designed, study coordination and drafting the manuscript; PGC: data collection, data analysis and drafting the manuscript; LGP: data collection and data analysis; PRC: data analysis and interpretation; RCGA, JCBM and JB: interpretation of data and critical review of the manuscript. All authors read and approved the final manuscript.
